# Sleep quality and incidence of diabetes in 0.5 million Chinese adults

**DOI:** 10.1186/s12889-025-25993-y

**Published:** 2025-12-19

**Authors:** Chunxiao Xu, Neil Wright, Yiping Chen, Pei Pei, Ling Yang, Dianjianyi Sun, Maxim Barnard, Jun Lv, Zhimin Gong, Canqing Yu, Junshi Chen, Weiwei Gong, Liming Li, Min Yu, Zhengming Chen, Meng Wang, Huaidong Du

**Affiliations:** 1 Department of Chronic Non-Communicable Diseases Control and Prevention, Zhejiang Provincial Center for Disease Control and Prevention, Hangzhou, No.630 Xincheng Road China; 2 Nuffield Department of Population Health (NDPH), University of Oxford, Clinical Trial Service Unit and Epidemiological Studies Unit (CTSU), Old Road Campus, Oxford, OX3 7LF UK; 3 Department of Epidemiology and Biostatistics, School of Public Health, Peking University, Beijing, China; 4 Key Laboratory of Epidemiology of Major Diseases (Peking University), Ministry of Education Beijing, Beijing, China; 5 Peking University Centre for Public Health and Epidemic Preparedness & Response, Beijing, China; 6 Pengzhou People’s Hospital, Chengdu, China; 7 China National Centre for Food Safety Risk Assessment, Beijing, China

**Keywords:** Diabetes mellitus, Prospective study, Sleep quality, China

## Abstract

**Background:**

Poor sleep quality is comparable to traditional risk factors for diabetes. This study evaluated the association between sleep quality index (SQI) and the risk of diabetes incidence in China.

**Methods:**

We used data from the China Kadoorie Biobank, comprising 462,743 participants aged 30 to 79 years without diabetes at baseline, who were recruited between 2004 and 2008 from ten diverse regions across China. We developed a SQI to assess sleep quality, based on seven self-reported sleep behaviors. The association of SQI (a lower value reflects a better sleep quality) on diabetes risk was assessed using Cox proportional hazards models, adjusting for a range of covariates including mainly potential risks factors of diabetes, i.e., waist circumference, body mass index, frailty, and poor health.

**Results:**

During an average follow-up of 11.7 years, a total of 18,397 incident cases of diabetes were identified among study participants. Compared with individuals with a SQI of 0, those with SQI of 1, 2, 3, or ≥ 4 had adjusted hazard ratio of 1.07 [95% confidence interval: 1.05–1.10], 1.17 (1.13–1.21), 1.12 (1.06–1.19), and 1.28 (1.17–1.39), respectively. Additionally, each one-unit higher SQI was associated with 6% higher risk. The association was very consistent between sexes and across various subgroups. Our study also found that sleep quality is closely linked to various socioeconomic and lifestyle factors, including education, occupation, levels of physical activity, sedentary habits, and adiposity.

**Conclusions:**

Poor sleep quality was a significant risk factor of developing diabetes in Chinese adults from both rural and urban areas. Promoting a healthy sleeping habit may be a promising public health strategy for the prevention of diabetes.

**Supplementary Information:**

The online version contains supplementary material available at 10.1186/s12889-025-25993-y.

## Introduction

The prevalence of diabetes has significantly grown over the past three decades, with approximately 592 million people globally predicted to be affected by 2035 [1]. China has a high prevalence of diabetes, with a total population living with the disease being as high as 116.4 million [2]. To mitigate the negative impact of diabetes, a more in-depth understanding of its complex disease pathogenesis and identification of modifiable risk factors are crucial [3].

In recent years, as life speeds up, people are experiencing sleep deprivation, with about 15% of the population suffering from insomnia [4]. Research has shown that sleep disturbances, including insomnia symptoms, can significantly influence multiple facets of physiological functioning, including endocrinology, immunology, and metabolism. There is growing scientific evidence suggesting that extreme sleep duration can increase the risk of developing diabetes in adults [5]. For example, a U-shaped association between sleep duration and incident diabetes was shown in several studies [6], suggesting that both abbreviated and extended length of sleep are linked to a likelihood of developing diabetes. However, such conclusion has not been consistently drawn by other studies [7, 8]. In addition, a meta-analysis of pooling six prospective cohort studies (40,649 participants) revealed a strong association between having difficulty initiating or maintaining sleep and the risk of developing diabetes [9]. Kowall et al. reported that waking up early was significantly associated with a higher risk of diabetes over a 5-year follow-up period [10]. Nilsson et al. found that individuals who experienced difficulty initiating sleep or used sleeping medication had a 52% higher risk of developing diabetes [11]. One study observed that daytime dysfunction, a sign of insufficient good sleep, was associated with increased severity of diabetic retinopathy [12]. On the other hand, napping has usually been regarded as a healthy lifestyle practice, particularly in Chinese populations with a siesta culture. However, this perspective has been increasingly questioned in recent years, as some study findings have suggested that napping may be associated with higher risk of diabetes [13, 14]. Moreover, our own previous work found that snoring was independently associated with a higher risk of diabetes in Chinese adults [15].

Most of these afore-mentioned studies examined the relationship of one or two sleep-related features with risk of diabetes in a relatively small number of participants. Therefore, evidence is still lacking about whether overall sleep quality is associated with risk of diabetes. Sleep quality is generally evaluated based on the depth of sleep (i.e., frequency of waking at night), sleep duration, and sleep-related effects on daytime performance (i.e., daytime dysfunction) [16, 17]. No consensus has been reached regarding its association with diabetes risk [16, 18–20], due potentially to the heterogenous definitions used to define sleep quality and small sample sizes of the association studies.

Therefore, the aim of the present study was to investigate the independent associations between a sleep quality index (SQI) and the incidence of diabetes among Chinese adults, using cohort data from the China Kadoorie Biobank (CKB) study, which includes approximately half a million participants.

## Methods

### Study design and study population

The methodology and details of the CKB study design and population have been previously discussed in other sources [21]. Overall, we have selected 10 diverse regions across China, where a comprehensive baseline survey was conducted from 2004 to 2008, successfully recruiting 512,724 adults aged 30–79 years. A laptop-based questionnaire was used to collect data on sociodemographic characteristics, tobacco use, alcohol intake, eating habits, physical activity, general health (including disease history and current medication use), familial history of disease (such as diabetes, cardiovascular diseases and malignant tumors). Calibrated instruments were used to measure anthropometric and physical parameters of participants according to standardized protocols, including height, weight, waist circumference, and blood pressure. Random plasma glucose (RPG) was assessed using the SureStep Plus meter (LifeScan, Milpitas, CA, USA) [22]. Individuals who reported a history of physician-diagnosed diabetes were classified as having self-reported diabetes. Those without self-reported diabetes but had on-site measured RPG level of 7.8–11.0 mmol/L were tested for fasting glucose the next day. Participants (those without self-reported diabetes) were defined as screen-detected diabetes if any of the following criteria was met: an RPG level ≥ 7.0 mmol/L after more than 8 h since the last meal; an RPG level ≥ 11.1 mmol/L within 8 h of the last meal; or a fasting plasma glucose level ≥ 7.0 mmol/L on subsequent testing [22]. Participants with either self-reported diabetes or screen-detected diabetes at baseline were excluded from the current analysis.

### Assessment of sleep-related information

For information on duration of sleep, the following question was asked during questionnaire survey: “How many hours do you sleep during a 24-hour period?” The answers included both nighttime sleep and daytime naps [23]. For daytime napping, participants were asked “do you usually take a daytime nap?” and the answer options included “Yes, usually”, “Yes, but only in summer”, and “No”. To measure snoring frequency, participants were asked to answer the question “Do you snore during sleep?” by selecting one answer from the following options: “Frequently”, “Sometimes”, and “Never”. To assess insomnia, participants were asked whether they have experienced the following problems for at least 3 days a week during the past year: had difficulty initiating or maintaining sleep, experienced early morning awakening, needed to use sleeping medication, or struggled to maintain a clear mind during the day due to poor sleep [24]. According to the fifth version of Diagnostic and Statistical Manual of Mental Disorders diagnostic criteria for insomnia, participants who answered yes to any of the above four questions were defined as having insomnia. SQI was calculated by summing up all scores from the above mentioned seven sleep behaviors: extreme sleep duration (1 if ≤ 6 h or ≥ 9 h; 0 otherwise), habitual snoring (1 if frequently, 0 otherwise), frequent daytime naps (1 if usually, 0 otherwise), four insomnia symptoms as above-described (1 if yes, 0 otherwise). The sum of these components provides the global SQI, which may range from 0 to 7, with higher values indicating poorer sleep quality.

### Measurements of covariates

We included age, sex, region, smoking status, alcohol consumption status, education, physical activity, TV/reading hours, family history of diabetes, self-rated poor health, frailty status, waist circumference and body mass index (BMI) as covariates to address potential confounding. Physical activity was quantified using metabolic equivalent task hours (MET-hours/day), which summarizes the total activities performed at work, transportation, domestic chores, and non-sedentary recreation each day. Participants were identified as having a family history of diabetes if their parents, children, or siblings had been diagnosed with diabetes. Self-rated health was measured using the question “How is your current health status?”, with the response options including “excellent”, “good”, “fair”, and “poor”. Based on previous work in the CKB, the frailty index was calculated for each participant as the number of deficits present in a person divided by the 24 deficits considered (eTable 1), after excluding diabetes, cardiovascular disease (CVD) and sleep related deficits from the original 28 deficit frailty index [25]. We further categorized the frailty index into three levels of frailty status: robust (≤ 0.10), prefrail (> 0.10 to < 0.25), and frail (≥ 0.25).

### Follow‑up and endpoint definition

Participants were monitored for morbidities and mortality attributed to specific causes through linkages with death (i.e., the Disease Surveillance Points System) and chronic disease registries as well as the nationwide health insurance hospitalization database, which has nearly comprehensive coverage (~ 99%) of our study population [21]. In addition, participant’s vital status was carefully checked against residential records through active follow-up involving participants’ family members as well as street committee or village administrators.

Both fatal and nonfatal events were coded using the International Classification of Diseases, 10th Revision (ICD-10), by staff members who were blinded to the baseline information. The primary outcome of this analysis was incident diabetes, E10-E14. The follow-up time of each individual participant was calculated as the time from the study entry to the date of diabetes diagnosis, death, lost to follow-up (0.8% in the entire CKB cohort), or the global censoring date which was 31 st Dec 2018, whichever occurred first.

### Statistical analysis

In addition to individuals with prevalent diabetes at baseline (*n* = 30,300), we also excluded those with missing BMI data (*n* = 2), a history of cardiovascular disease (CVD) (*n* = 19,244), or implausible/conflicting sleep information—such as reporting ≥ 10 h per day of sleep alongside both difficulty initiating sleep and early awakening (*n* = 433), or reporting zero hours of sleep (*n* = 2). A total of 462,743 participants remained in the current analysis (eFigure 1).

Continuous variables were expressed as mean ± standard deviation (SD), while categorical variables were reported as percentage for describing population characteristics by SQI categories. To understand the patterns of SQI by age, sex, region, social economic status (i.e., education and occupation) lifestyle factors (i.e., physical activity, TV/reading hours) and adiposity status (i.e., BMI and waist circumference), multiple linear regression analyses were performed with SQI (as a continuous variable) as a dependent variable and adjusting for age (continuous), sex and region (10 areas), where appropriate.

We examined the cross-sectional associations of SQI (5 categories as 0, 1, 2, 3, or ≥ 4) with physical measures, including systolic blood pressure (SBP), diastolic blood pressure (DBP), pulse pressure (PP), and RPG, using multiple linear regression analyses with adjustment for age, sex and region. Logistic regression analysis was conducted to assess the odds ratios (ORs) of baseline prevalence of frailty and poor self-rated health across 5 categories of SQI, adjusting for age, sex, region, education level (primary school and below, middle school or higher), smoking status (never, occasional, or current regular), alcohol intake (never, occasional, or current regular), TV/reading hours (continuous), total physical activity (continuous), family history of diabetes (yes/no), BMI (decile), and waist circumference (decile).

Cox regression was used to calculate adjusted hazard ratios (HRs) for incident diabetes across categories of SQI. Proportional hazards assumption was tested using the Schoenfeld residual method and no violation was found. Four distinct models were built, with model 1 including only sex, age-at-risk (in 5-year interval) and region as strata variable to control potential confounding without assuming proportionality of these variables, model 2 additionally adjusting for smoking status, alcohol intake, education, physical activity, TV/reading hours, and family history of diabetes, model 3 additionally adjusting for poor self-rated health status (yes/no) and frailty status (yes/no), and finally model 4 also adjusting for waist circumference (deciles) and BMI (deciles). These covariates were selected because they are potential risk factors of diabetes and correlates of SQI according to previous literature and our own results. Multicollinearity was checked using the adjusted generalized standard error inflation factor and no violation was found. Similar Cox regression analysis was also conducted to explore the association between diabetes and each individual component of SQI, including sleep duration (≤ 5, 6, 7, 8, 9, or ≥ 10 h), habitual snoring (3 categories), frequent daytime napping (3 categories), insomnia (yes/no) and 4 insomnia symptoms (yes/no), with mutual adjustment for all other components of SQI. For exposures with more than 2 categories, the floating absolute risk method was used to estimate standard errors of log HRs for all exposure categories, including the reference category [26]. Sensitivity analysis was performed with exclusion of the first two years of follow-up.

All statistical analyses were conducted utilizing the SAS analytical software version 9.4 from the SAS Institute Inc (Cary, NC, USA) and the R version 4.4.3 (The R Foundation for Statistical Computing). All figures were created using R. Statistical significance was determined if the two-sided *P* value was below 0.05.

## Results

### Characteristics of study participants

Among the 462,743 participants included in the analysis, the mean (SD) age of our study population was 51.2 (10.5) and 59.0% were women (Table 1). Overall, 42.3% participants resided in urban regions and 49.4% received middle school or above education. As presented in the eFigure 2, the prevalence of good sleep (i.e., SQI = 0) was higher in rural (45.7%) than in urban (37.3%) areas. The overall distribution of SQI was right-skewed (eFigure 3), with 42.1% participants scored 0 on the SQI, 36.1% scored 1, and 21.8% scored 2 or higher. The top three most prevalent components of SQI are extreme sleep duration (24.0%), habitual snoring (21.2%), and frequent naps (19.6%) (eFigure 4). As the SQI got higher (i.e., sleep quality became worse), the mean age was higher, physical activity level became lower, and the prevalence of poor health, frailty, and family history of diabetes became dramatically higher (Table 1).

**Table 1:** Baseline characteristics of study participants by sleep quality index

Characteristic variables	Sleep quality index					Overall
	0	1	2	3	≥ 4	
Participants, *n* (%)	194,936	167,013	67,949	23,196	9,649	462,743
Mean age (SD), years	49.9 (10.2)	51.4 (10.5)	53.1 (10.5)	54.6 (10.4)	55.5 (10.3)	51.2 (10.5)
Women, %	62.2	56.4	53.8	60.6	66.8	59.0
Living in urban area, %	37.3	44.8	48.1	46.0	49.2	42.3
Middle school or above, %	48.9	49.5	49.3	47.8	48.5	49.4
In marriage, %	91.0	91.4	90.9	89.4	88.1	91.1
Annual household income > 19,999 yuan, %	41.9	42.9	42.5	39.9	38.2	42.6
Current regular smoker, %						
Men	63.2	62.5	62.3	62.6	59.6	62.5
Women	2.1	2.3	2.4	2.8	2.5	2.2
Current regular drinker, %						
Men	32.7	34.5	36.1	35.8	33.6	34.0
Women	1.9	2.3	2.3	2.4	2.7	2.1
BMI (SD), kg/m^2^	23.2 (3.2)	23.7 (3.4)	23.9 (3.5)	23.7 (3.6)	23.5 (3.5)	23.5 (3.3)
Waist circumference (SD), cm	78.9 (9.0)	80.2 (9.7)	80.8 (10.1)	80.3 (10.2)	79.6 (10.1)	79.7 (9.6)
Systolic blood pressure (SD), mmHg	129.0 (20.1)	130.6 (21.0)	131.3 (21.6)	130.6 (21.7)	129.7 (21.6)	130.0 (20.8)
Diastolic blood pressure (SD), mmHg	76.8 (10.8)	77.8 (11.2)	78.4 (11.4)	78.2 (11.4)	77.8 (11.2)	77.5 (11.1)
Mean Physical activity (SD), MET-h/day	22.2 (14.0)	21.7 (13.9)	21.2 (13.6)	20.9 (13.2)	20.4 (12.8)	21.8 (13.9)
Sleep duration (SD), h/day	7.3 (0.7)	7.7 (1.3)	7.6 (1.8)	6.5 (2.2)	5.5 (1.7)	7.4 (1.4)
Tv/reading hours (SD), h/week	20.7 (10.3)	21.1 (10.7)	21.5 (11.2)	21.6 (11.6)	21.4 (11.8)	21.0 (10.7)
Study date at winter, %	20.0	20.6	20.9	21.1	20.7	20.3
Poor health, %	6.7	8.3	11.9	17.4	26.7	8.9
Frailty, %	2.0	2.8	4.0	6.0	9.9	3.0
Prevalence of hypertension, %	28.6	32.4	34.6	33.5	32.5	31.2
Family history of diabetes, %	5.6	6.2	6.9	7.1	7.6	6.1

Values were standardized according to age, sex and regions

*BMI* Body mass index, *SD* Standard deviation

### Patterns and correlates of SQI

**Fig. 1:**
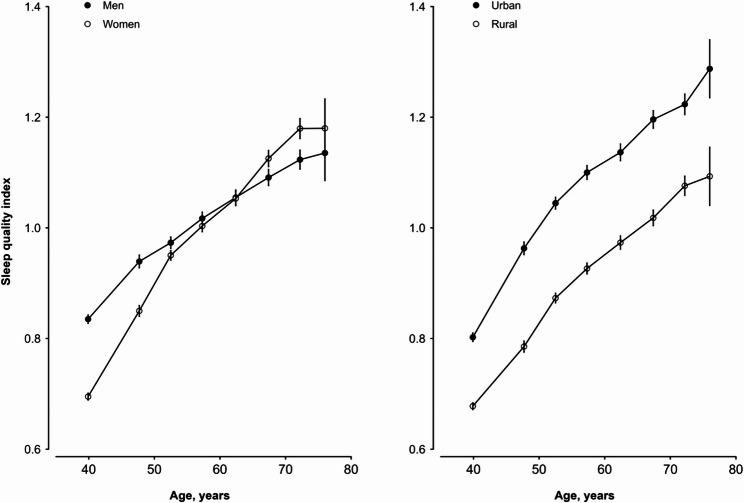
Distribution of sleep quality index by age, sex and region. Mean values (95% CIs) of the sleep quality index were adjusted for 10 regions in the left panel and for sex in the right panel. The mean sleep quality index was 1.09 in urban areas and 0.92 in rural areas. Abbreviations: CI: confidence interval

SQI became higher with increasing age, regardless of sex and rural/urban area of residence (Fig. 1). The speed of increase with age was more pronounced in women than in men. On average, the SQI was slightly higher in men (than in women) among those younger than 65 years old, but was slightly higher in women among those older than 65 years old. Overall, urban participants had an approximately 0.17-unit higher average SQI than rural participants across the entire age range observed.

**Fig. 2:**
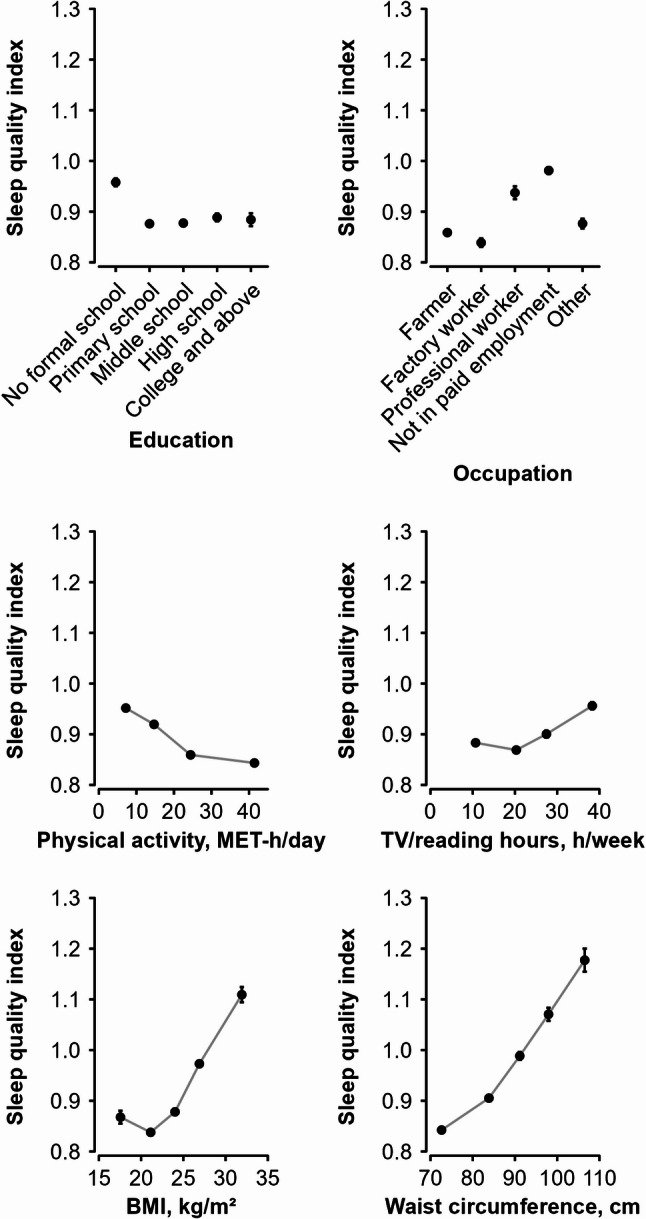
Sleep quality index by socio-economic and lifestyle correlates. Mean values (95% CIs) of the sleep quality index were adjusted for age, sex, and region. The mean sleep quality index was 0.87 in the highest category of total physical activity (40.9 MET-h/day) and 0.97 in the lowest category (6.4 MET-h/day). Across BMI categories, the mean sleep quality index was 1.13 in the highest category and 0.89 in the lowest. Similarly, in waist circumference categories, the corresponding values were 1.20 and 0.86, respectively. Abbreviations: CI: confidence interval, BMI: body mass index, MET-h/day: metabolic equivalent of task-hours per day

Participants with no formal school education had the highest mean SQI among all the groups with different levels of education, but no clear difference or trend was observed across the other 4 groups from primary school to college or higher education (Fig. 2). Among the different occupation categories, factory workers and farmers had lower mean SQI (i.e., better sleep), while those not in paid employment (i.e., unemployed, housewife or house husband) and those with professional or management jobs had higher SQI (i.e., poorer sleep). Physical activity level was inversely but sedentary leisure time was positively associated with mean SQI. Comparing the highest with lowest physical activity category (40.9 vs. 6.4 MET-h/day), the mean SQI was 0.10 unit lower. A similar difference in SQI was observed between the lowest and highest categories of sedentary leisure time, despite the value in the highest category was higher.

Both BMI and waist circumference were positively associated with SQI (Fig. 2), although the associations were slightly non-linear, i.e., the lowest SQI was observed among those with BMI between 18.5 and 23 kg/m^2^, which was significantly lower than those with BMI < 18.5 kg/m^2^. Comparing with those having the lowest degree of adiposity (i.e., BMI < 23 kg/m^2^ and waist circumference < 80 cm), those having highest degree of adiposity (i.e., BMI ≥ 30 kg/m^2^ or waist circumference ≥ 102 cm) had 0.24/0.34-unit higher SQI. No clear association was observed of other lifestyle factors (e.g., smoking, alcohol consumption, tea consumption and dietary intake) with SQI (data not shown).

### Cross-sectional association between SQI and health-related traits

**Fig. 3:**
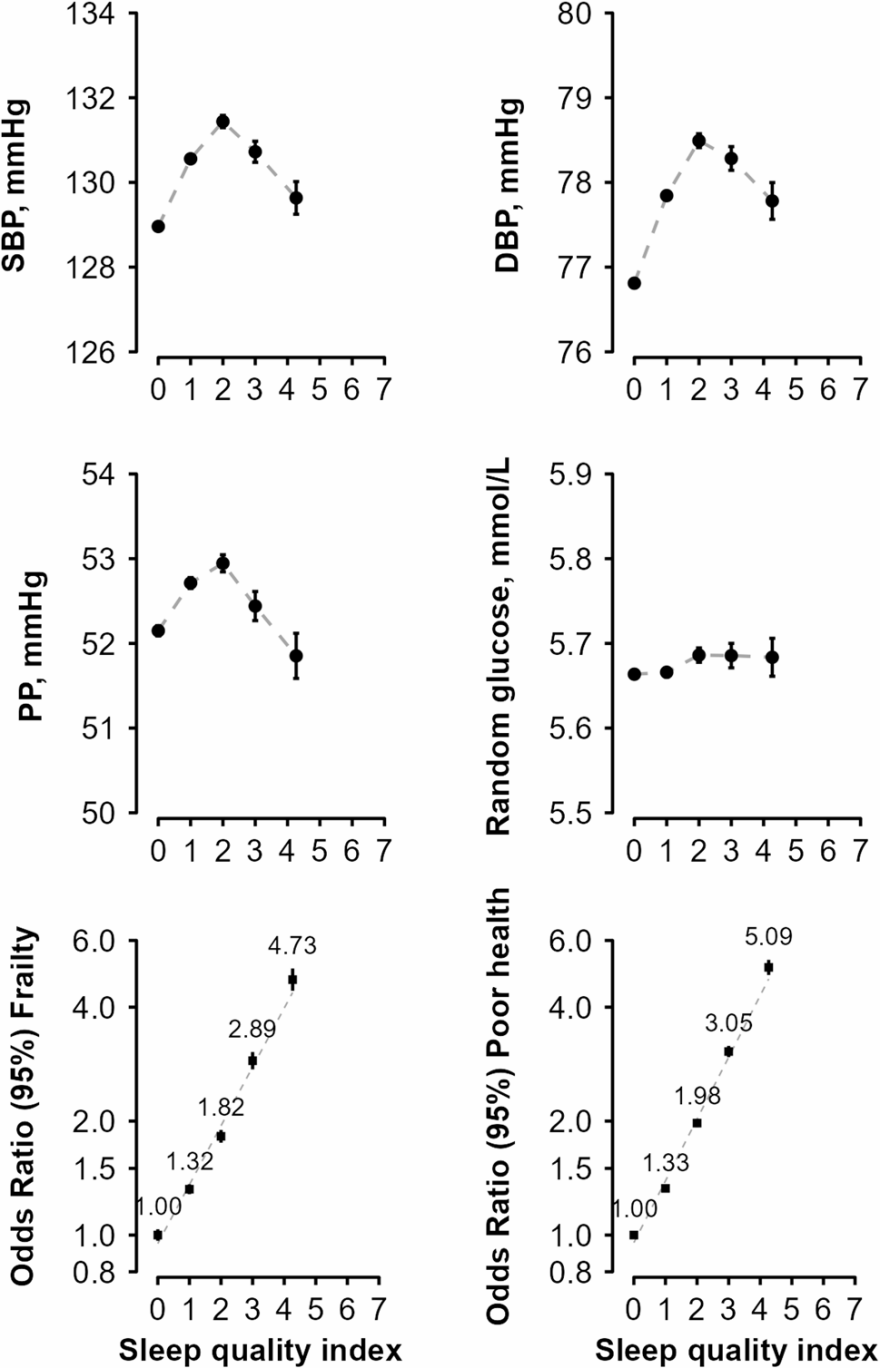
Sleep quality index in relation to cardiometabolic risk factors and baseline health status. Mean values (95% CIs) of SBP, DBP, PP and RPG were adjusted for age, sex and region. Odds ratios for frailty and poor health were adjusted for age, sex, region, education, smoking status, alcohol intake, TV/reading hours, total physical activity, family history of diabetes, BMI, and waist circumference. Frailty and poor health were mutually adjusted in the analyses. Abbreviations: SBP: systolic blood pressure, DBP: diastolic blood pressure, PP: pulse pressure

SQI was in a non-linear relationship with SBP, DBP and PP, with the lowest values of SBP and DBP observed among those with the best quality of sleep, i.e., lowest SQI (= 0) (Fig. 3). No clear association was observed between SQI and RPG. However, a strongly positive association was observed for SQI with frailty and self-rated poor health status. Compared to individuals with a SQI of 0, those with scores of 1, 2, 3, and ≥ 4 had progressively higher risks of frailty (adjusted ORs: 1.32 [1.26–1.38], 1.82 [1.72–1.92], 2.89 [2.67–3.04], and 4.73 [4.32–5.03], respectively) and poor health (1.33 [95% CI: 1.29–1.36], 1.98 [1.91–2.03], 3.05 [2.90–3.14], and 5.09 [4.80–5.30], respectively).

### Prospective association between SQI and incident diabetes

**Fig. 4:**
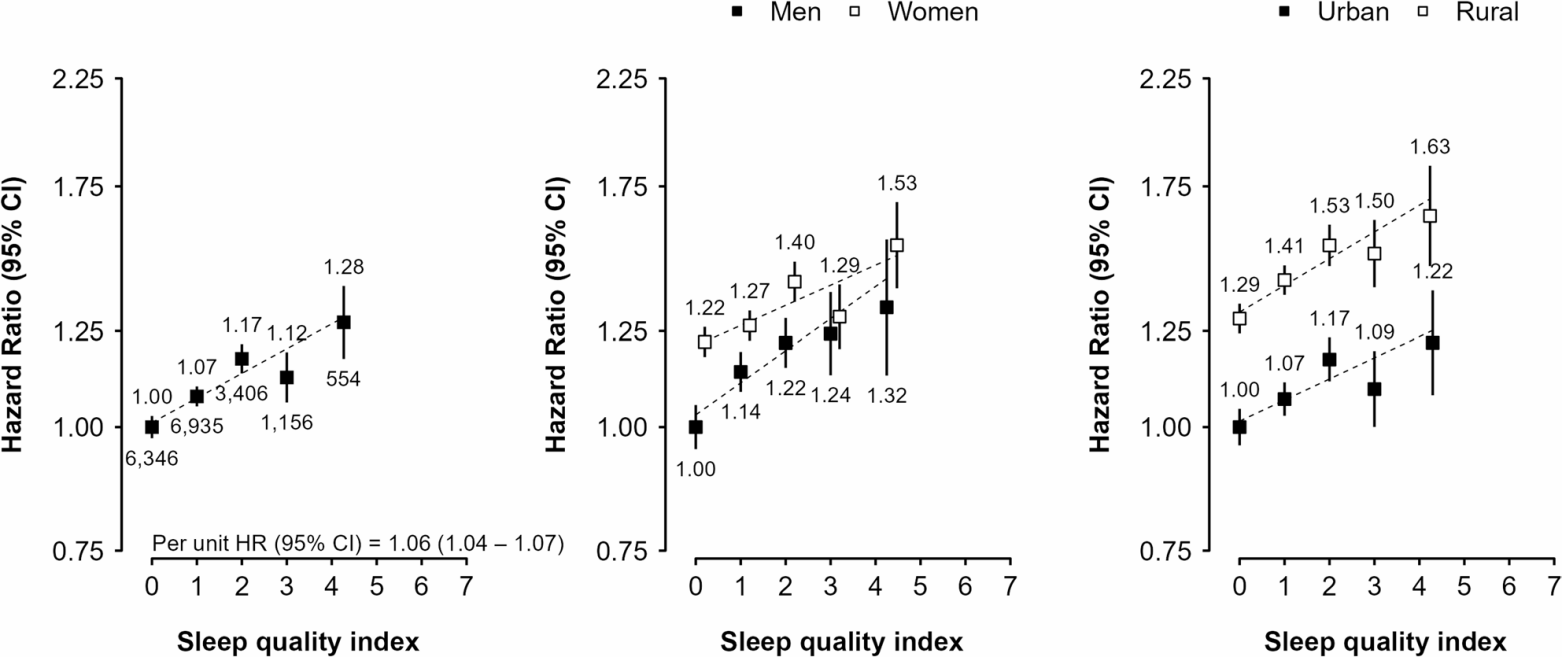
Adjusted HRs of diabetes mellitus for sleep quality index in overall, between men or women and urban or rural. The adjusted HRs (95% CI) were stratified by age-at-risk (5-year groups), sex, and region and were adjusted for education, smoking status, alcohol intake, TV/reading hours, total physical activity, family history of diabetes, frailty status, self-rated health, BMI, and waist circumference. The numbers shown below the CIs in the first panel were the number of events. Abbreviations: HR: hazard ratio, CI: confidence interval

A total of 18,397 incident diabetes cases were recorded during 5.4 million person-years of follow up (mean follow up duration of 11.7 years). Compared with individuals having a SQI of 0, those with SQI of 1, 2, 3, or ≥ 4 had a largely consistently higher absolute risk of diabetes, with adjusted HRs of 1.07 (95% CI: 1.05–1.10), 1.17 (1.13–1.21), 1.12 (1.06–1.19), and 1.28 (1.17–1.39), respectively, after controlling for all potential confounders in the final model (Table 2). Among the components of SQI, snoring had the strongest association with diabetes incidence, showing 18% (15% − 22%) higher risk in those having a habitual snoring habit than those do not snore. In addition, frequent napping was also independently associated with diabetes risk, with HR being 1.11 (1.07–1.15) in the fully adjusted model (i.e., model 4) comparing to those who has no daytime napping habit. In contrast, participants with insomnia, difficulty initiating sleep and daytime dysfunction had a slightly but statistically significant lower risk of diabetes in the model 3. But these relationship disappeared after further adjustment for adiposity (Table 2). Similarly, in the initial model (Model 1), longer sleep duration of ≥ 10 h per day was significantly associated with a slightly elevated risk of diabetes compared to those reported 7 h per day of sleep, with HR being 1.13 (1.05–1.22). However, this association was attenuated and became non-significant after further adjustments for additional covariates. The strength of association between SQI and diabetes incidence was similar across sexes and in rural and urban areas (Fig. 4). Overall, each one-unit increase in the SQI was associated with a 6% higher risk of diabetes.

**Table 2:** Hazard ratios of sleep related traits with incidence of diabetes

Sleep-related traits	Cases, *n*	Person-years	Incidence rate, per 10,000 person-years			HR (95% CI)	
				Model 1	Model 2	Model 3	Model 4
Sleep quality index							
0	6,346	2,282,063	27.8	1.00 (0.97–1.03)	1.00 (0.97–1.03)	1.00 (0.97–1.03)	1.00 (0.97–1.03)
1	6,935	1,945,263	35.7	**1.24 (1.22–1.27)**	**1.23 (1.20–1.26)**	**1.17 (1.14–1.19)**	**1.07 (1.05–1.10)**
2	3,406	784,402	43.4	**1.45 (1.41–1.50)**	**1.42 (1.37–1.47)**	**1.29 (1.25–1.34)**	**1.17 (1.13–1.21)**
3	1,156	266,424	43.4	**1.37 (1.29–1.45)**	**1.33 (1.26–1.41)**	**1.18 (1.11–1.25)**	**1.12 (1.06–1.19)**
≥ 4	554	110,819	50.0	**1.56 (1.44–1.70)**	**1.50 (1.38–1.63)**	**1.27 (1.17–1.39)**	**1.28 (1.17–1.39)**
Sleep duration, hour ^a^							
≤ 5	1,699	415,283	40.9	1.01 (0.95–1.06)	1.05 (0.99–1.12)	1.03 (0.97–1.09)	1.03 (0.97–1.10)
6	2,732	760,751	35.9	0.98 (0.93–1.03)	0.99 (0.95–1.04)	0.99 (0.94–1.04)	0.99 (0.94–1.04)
7	4,661	1,351,590	34.5	1.00 (0.96–1.04)	1.00 (0.96–1.04)	1.00 (0.96–1.04)	1.00 (0.96–1.04)
8	6,363	1,981,538	32.1	1.02 (0.98–1.06)	1.01 (0.97–1.05)	1.02 (0.98–1.06)	1.01 (0.98–1.05)
9	2,057	605,957	33.9	1.08 (1.02–1.13)	1.04 (0.98–1.09)	1.02 (0.97–1.08)	1.03 (0.98–1.09)
≥ 10	885	273,851	32.3	**1.13 (1.05–1.22)**	1.08 (1.01–1.16)	1.05 (0.98–1.13)	1.08 (1.00–1.16)
Snoring ^a^							
None	7,615	2,962,654	25.7	1.00 (0.98–1.02)	1.00 (0.98–1.02)	1.00 (0.98–1.02)	1.00 (0.98–1.02)
Sometimes (Occasional)	4,604	1,300,252	35.4	1.27 (1.24–1.31)	1.25 (1.22–1.29)	1.21 (1.17–1.24)	1.03 (1.00–1.06)
Usually (Habitual)	6,178	1,126,065	54.9	**1.83 (1.78–1.88)**	**1.78 (1.73–1.82)**	**1.61 (1.57–1.65)**	**1.18 (1.15–1.22)**
Daytime napping ^a^							
None	7,818	2,136,868	36.6	1.00 (0.98–1.02)	1.00 (0.98–1.02)	1.00 (0.98–1.02)	1.00 (0.98–1.02)
Summer-only	6,836	2,209,296	30.9	**1.05 (1.02–1.08)**	1.02 (0.99–1.04)	1.02 (0.99–1.04)	1.01 (0.98–1.03)
Usually (Frequent)	3,743	1,042,807	35.9	**1.25 (1.21–1.30)**	**1.18 (1.14–1.23)**	**1.16 (1.11–1.20)**	**1.11 (1.07–1.15)**
Insomnia ^a^							
No	15,125	4,519,635	33.5	1.00	1.00	1.00	1.00
Yes	3,272	869,337	37.6	0.97 (0.93–1.01)	0.98 (0.94–1.02)	**0.92 (0.88–0.96)**	1.01 (0.97–1.06)
Insomnia parameters							
Difficulty initiating sleep ^a^							
No	16,215	4,790,351	33.8	1.00	1.00	**1.00**	1.00
Yes	2,182	598,620	36.5	0.99 (0.94–1.03)	1.01 (0.96–1.06)	**0.94 (0.90–0.99)**	1.02 (0.97–1.08)
Waking up too early ^a^							
No	16,239	4,842,099	33.5	1.00	1.00	1.00	1.00
Yes	2,158	546,872	39.5	0.99 (0.95–1.04)	1.00 (0.95–1.06)	0.95 (0.91–1.00)	1.05 (0.99–1.10)
Daytime dysfunction ^a^							
No	17,963	5,270,160	34.1	1.00	1.00	1.00	1.00
Yes	434	118,811	36.5	1.01 (0.92–1.12)	1.00 (0.90–1.10)	**0.90 (0.81–0.99)**	0.99 (0.90–1.09)
Sleep needing medicine ^a^							
No	18,143	5,331,041	34.0	1.00	1.00	1.00	1.00
Yes	254	57,931	43.8	1.14 (1.01–1.29)	1.11 (0.98–1.25)	0.99 (0.87–1.13)	1.10 (0.97–1.25)

Model 1: Stratified by age-at-risk, sex and regions

Model 2: Additionally adjusted for smoking, current drinking, education, physical activity, family history of diabetes, TV/reading hours

Model 3: Additionally adjusted for frailty status and poor self-rated health

Model 4: Additionally adjusted for waist circumference and BMI

^a^ Analyses of sleep duration, habitual snoring, daytime napping, and insomnia were mutually adjusted for one another

**Fig. 5:**
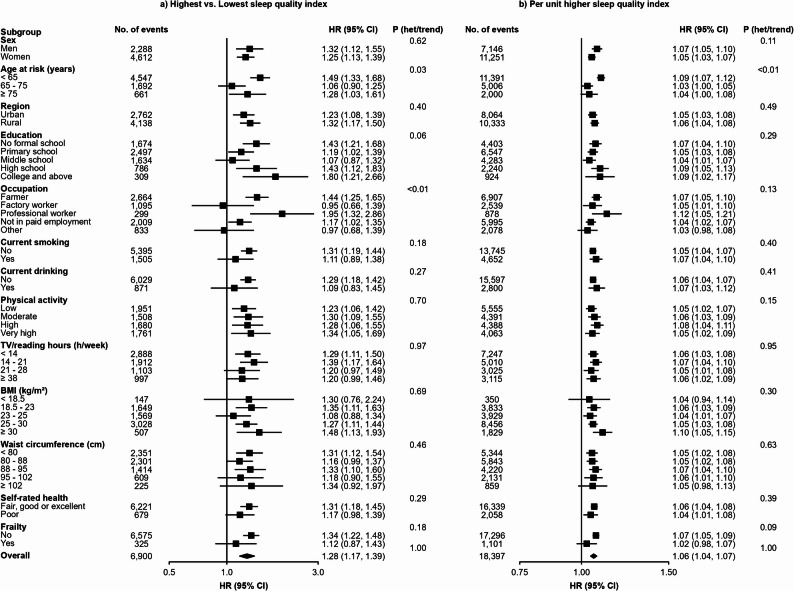
Sleep quality index in relation to risk of diabetes in various subgroups. The adjusted HRs (95% CI) were stratified by age-at-risk (5-year groups), sex, and region and were adjusted for education, smoking status, alcohol intake, TV/reading hours, total physical activity, family history of diabetes, frailty status, self-rated health, BMI, and waist circumference. Abbreviations: CI: confidence interval, BMI: body mass index, P het/trend: P value for heterogeneity test or trend test

In subgroup analysis stratified by age-at-risk, sex, region, education level, occupation, status of current smoking and drinking, physical activity, TV/reading hours, BMI, waist circumference, self-rated health and frailty status, no heterogeneity was observed (*P*_*for bonferroni*_ > 0.05/13) (Fig. 5). In the sensitivity analysis, the results were not essentially changed after excluding individuals who developed diabetes within the first 2 years of follow up (eTable 2).

## Discussion

In the current analysis of data from a prospective cohort study of nearly 0.5 million Chinese adults, we found that poor sleep quality was significantly associated with higher risk of diabetes. Compared to a SQI score of 0, those without any sleeping-related problem, adjusted HR for those with 1, 2, 3, or ≥ 4 sleeping-related problem(s) was 1.07, 1.17, 1.12, and 1.28, respectively. On average, each one-unit higher SQI (indicating one additional sleep-related problem) was associated with a 6% higher risk for developing diabetes.

Our study also revealed several notable correlations between sleep quality and various socioeconomic and lifestyle factors, including education, occupation, physical activity, sedentary time and adiposity. People with no formal education exhibited the poorest sleep quality, potentially related to socioeconomic stress, limited health literacy, and reduced access to healthcare—factors known to negatively affect sleep hygiene and overall well-being. Physical activity, widely recognized as a non-pharmacological intervention for neurological, metabolic, and cardiovascular conditions, was associated with better sleep in our study. Individuals with higher physical activity levels, such as factory workers and farmers, reported better sleep quality (i.e., lower SQI) than those in less physically demanding occupations. Conversely, extended sedentary behaviors like prolonged TV viewing or reading were linked to poorer sleep quality.

Existing evidence in general supports that overweight and obesity are interlinked with poor sleep quality. For instance, a study involving 1,578 college students in the U.S. and Korea found that obesity was linked to both inappropriate sleep duration and reduced sleep quality [27]. In another study of 161 individuals with weight status and sleep quality assessed three months apart, it was found that higher adiposity was associated with poorer sleep quality independently of co-occurring behaviors such as inadequate physical activity and alcohol consumption [28]. Consistent with these findings, our current study found that higher BMI (> 23 kg/m^2^) and greater waist circumference were associated with poorer sleep quality.

We did not observed any clear mono-modal dose-response association of SQI with SBP, DBP, PP or RPG in the current study, although those participants with the best quality sleep had significantly lower values of DBP. These findings are inconsistent with previous results tended to suggest a potential beneficial role of good quality sleep in glucose metabolism and blood pressure control [29, 30]. The non-significant association between SQI and RPG in our study might be at least partially related to the low statistical power due to small variation in RPG among non-diabetic participants at baseline. The no clear association of SQI with these metabolism-related measures in our study warranted future intervention studies with objectively measured sleep quality and closely monitored metabolism status.

The clear and strongly positive associations of SQI with prevalence of frailty and poor self-rated health status underscore the potential benefits of a good sleep for overall well-being. These findings are consistent with previous studies showing that poor sleep quality is associated with frailty [31–34] and poor overall health [35]. However, most of these previous studies, including the analyses we performed, were of cross-sectional design. Future studies are necessary to clarify the direction of associations, i.e., poor sleep leading to poor health or poor health influencing sleep quality.

Until now, only a few prospective studies with small sample size have investigated the association of sleep quality with the risk of developing diabetes [16, 18–20, 36], showing inconsistent results. Gilad et al. reported that abnormal sleep quality (highest quartile of sleep quality score vs. normal score), assessed using Mini-sleep Questionnaire, was associated with a 53% higher risk of diabetes and per point higher sleep quality score was associated with 3.6% higher risk of diabetes [18], slightly weaker than the 6% higher risk corresponding to per 1-unit SQI higher observed in our study. However, another study, performed in the Southall and Brent REvisited cohort, did not observe a significant association between poor sleep quality and incidence of diabetes. In this study sleep quality was comprised of having difficulty falling asleep, waking up too early, feeling tired on waking up and snoring [36].

Habitual snoring (but not occasional snoring) was found as the strongest sleep quality component in relation to diabetes incidence in the current study. This confirmed the finding from a previous analysis of CKB data showing that habitual snoring was independently linked to an increased risk of developing type 2 diabetes by over 10% [15]. In addition, a prospective study of 2,668 Swedish men aged 30 to 69 years [37] found that habitual snoring is associated with an increased incidence of diabetes within 10 years. And another prospective study conducted in 69,852 females in the United States [38] reported that both occasional and regular habitual snoring were associated with an increased risk of developing diabetes. Snoring is commonly linked to obstruction in the upper airways and may be an indicator of obstructive sleep apnea. The repeated episodes of low oxygen (hypoxia) and high carbon dioxide (hypercapnia) levels during sleep can trigger heightened sympathetic nervous system activity and increased oxidative stress, both of which contribute to insulin resistance [39]. Moreover, the production of proinflammatory cytokines plays a significant role in the development of diabetes related to snoring [40].

Previous evidence from prospective studies generally suggests a U-shaped or J-shaped association between sleep duration and diabetes risk [6], meaning that both inadequate and excessive amount of sleep could potentially increase the likelihood of developing diabetes. For instance, a 10-year follow-up survey of 70,026 U.S. women aged 30–55 years participating in the Nurses’ Health Study revealed that long sleep substantially increased the diagnosis of diabetes [41]. However, we only observed such a similar tendency in our least adjusted model. The higher risk of diabetes among those reported at least 10 h per day sleep (as compared to those reported 7 h of sleep per day) became borderline non-significant after adjustment for all potential confounders, suggesting that the association and underlying mechanisms between sleep duration and diabetes remain undetermined.

Daytime napping is often perceived as a healthy behavior and, as a result, has been excluded from sleep quality assessments in many previous studies. Actually, napping appears to play an important role in diabetes risk [42] and has also been linked to increased mortality [43]. McWhorter et al. [44] investigated the association between napping frequency and type 2 diabetes mellitus using individuals who napped less than three times per week as the reference group and found that frequent napping (≥ 3 times/week) was associated with a higher risk of type 2 diabetes mellitus. Similarly, Zhou et al. [45] analyzed data of 435,342 adults in the UK Biobank and reported that increased nap frequency was linked to a greater risk of type 2 diabetes mellitus. Our findings confirmed these previous results, suggesting frequent daytime napping may increase the risk of developing diabetes. While the underlying mechanisms remain unclear, daytime napping—often viewed as a compensatory response to sleep deprivation or poor sleep quality—has been associated with elevated levels of inflammatory markers, such as serum C-reactive protein, which may contribute to the development of diabetes [46].

Insomnia symptoms have reached epidemic proportions, affecting a substantial portion of the global population. Through a 5-year follow-up study, Kowall et al. found that waking up early can significantly increase the risk of diabetes [10]. However, neither the overall insomnia indicator (defined by the presence of any of the 4 insomnia systems) nor the 4 insomnia symptoms was significantly associated with a higher risk of diabetes in our study, suggesting the necessity of further study.

As a composite index measuring the overall quality of sleep, all these above-mentioned mechanisms linking individual sleep features with diabetes risk may play a role in linking SQI to diabetes risk. Studying sleep quality as a whole, rather than examining individual components in isolation, offers a more comprehensive and ecologically valid understanding of sleep-diabetes relationship. Sleep is inherently multidimensional, and the combined effects of various aspects—such as duration, continuity, snoring and napping—often interact to influence health outcomes. Composite measures of sleep quality, like a global SQI, better capture this complexity and may show stronger associations with diabetes. Additionally, analyzing sleep as an integrated construct can avoid issues of multicollinearity and multiple comparisons, enhance statistical power, and facilitate clearer interpretation. In contrast, focusing solely on individual components may underestimate the cumulative burden of poor sleep, limiting both scientific insight and practical application.

Our current study is a large, prospective, population-based cohort study with data covering diverse areas, increasing the likelihood that its findings can be generalized to broader Chinese population. In addition, we ensured that the data collection was comprehensive, and used robust case ascertainment methods through comprehensive follow-up systems to limit potential biases. We also broadened our adjustment for potential confounders, including some may have a mediating role at the same time (such as waist circumference, BMI, frailty and poor health), to reduce the risk of potential bias. Finally, the SQI in this study incorporates a comprehensive range of sleep behaviors, including daytime napping—rarely considered in other studies, offering a more holistic assessment of sleep patterns compared to studies that examine individual sleep components in isolation. This approach allows for a better understanding of how combined sleep disturbances may contribute to the risk of developing diabetes. Not a surprise, our study has limitations too. Firstly, the self-reported sleep parameters from a population-based survey can be influenced by subjectivity, and as such, the measurement errors in SQI could potentially bias (i.e., under estimate) the results. Secondly, the calculation of SQI was based on equal weight of all available sleep-related traits and validity of it has not been confirmed due to lack of golden standard. Thirdly, it should be noted that in our analysis a large portion of diabetes cases included in the current analysis were unspecified diabetes (E14) rather than type 2 diabetes. Given the fact that all our participants were above 30 years old at baseline (mean baseline age above 50 years), it is reasonable to assume that incident diabetes cases during follow-up are predominantly of type 2 diabetes. Finally, in spite of the fact that a comprehensive set of potential confounders had been considered, possibility of residual confounding from other known or unknown risk factors in this observational study could not be ruled out. Further intervention or genetic studies (e.g., Mendelian Randomization analysis) are needed to establish whether sleep quality has a causal role on diabetes incidence.

## Conclusion

Our data suggests that people with poor sleep quality are more likely to develop diabetes. Efforts to improve sleep quality may help to attenuate individuals’ risk of diabetes and reduce the overall diabetes burden in population.

## Supplementary Information

Supplementary Material 1.

## Data Availability

The datasets used and/or analyzed during the current study are available from the corresponding author on reasonable request. Public availability is restricted to protect participant privacy.

## Abbreviations

SQI: Sleep quality index
BMI: Body mass index
SBP: Systolic blood pressure
DBP: Diastolic blood pressure
PP: Pulse pressure
RPG: Random plasma glucose
CVD: Cardiovascular disease
CKB: China Kadoorie Biobank
HR: Hazard ratio
CI: Confidence interval
