# Supplementary material for: Sleep quality and incidence of diabetes in 0.5 million Chinese adults
Source: BMC Public Health. 2025 Dec 19;26:302. doi: 10.1186/s12889-025-25993-y (PMC12831405; doi:10.1186/s12889-025-25993-y)
Supplement: Supplementary file 1 — Supplementary Material 1. [file 12889_2025_25993_MOESM1_ESM.pdf]

Sleep quality and incidence of diabetes in 0.5 million Chinese adults

Supplementary material

eFigure 1.Flow chart showing the progression of the participants in the China Kadoorie Biobank (CKB) study

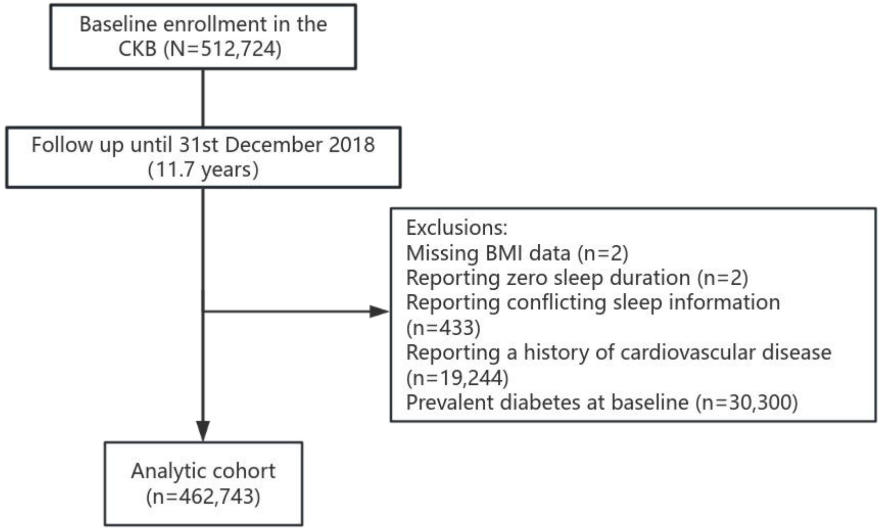

eFigure 2. Prevalence of good sleep by region

Values were adjusted for age and sex. Participants who reported a sleep quality index of 0 were classified as having good sleep

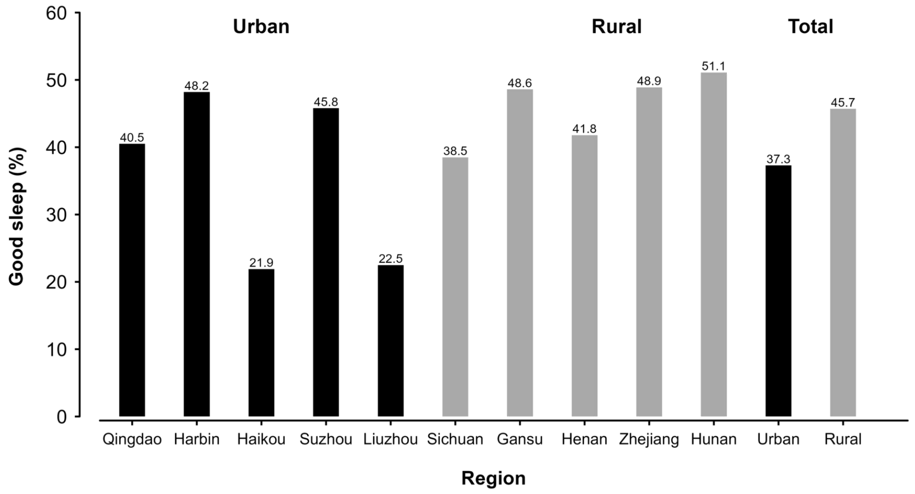

eFigure 3. Distribution curve of sleep quality index with trend line

Mean value of sleep quality index is 0.89.

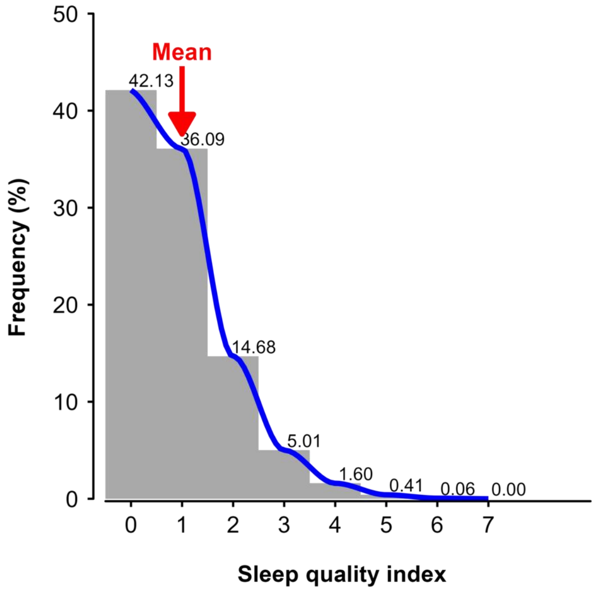

eFigure 4. Prevalence of sleep quality components

Values were adjusted for age and sex. Participants who reported a sleep quality index of 0 were classified as having good sleep.

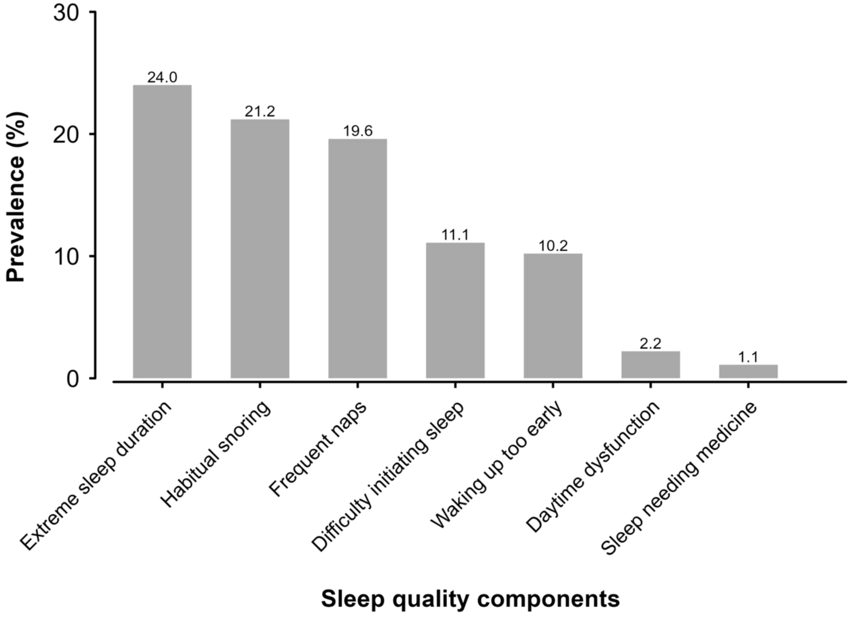

eTable 1. List of 24 variables included in the frailty index

	Definition according to baseline self-report, physical measurement, or both	Coding of variables
**1**	Self-reported diagnosis of hypertension by a doctor, self-reported use of antihypertension drugs, systolic blood pressure measured to be ≥ 140 mm Hg, or diastolic blood pressure measured to be ≥ 90 mm Hg	Yes=1.00; no=0.00
**2**	Self-reported diagnosis of emphysema or chronic bronchitis by a doctor	Yes=1.00; no=0.00
**3**	Self-reported diagnosis of tuberculosis by a doctor	Yes=1.00; no=0.00
**4**	Self-reported diagnosis of asthma by a doctor	Yes=1.00; no=0.00
**5**	Self-reported diagnosis of peptic ulcer by a doctor	Yes=1.00; no=0.00
**6**	Self-reported diagnosis of gallstone disease, with or without cholecystitis, by a doctor	Yes=1.00; no=0.00
**7**	Self-reported diagnosis of rheumatoid arthritis by a doctor	Yes=1.00; no=0.00
**8**	Self-reported diagnosis of fracture by a doctor	Yes=1.00; no=0.00
**9**	Self-reported diagnosis of neurasthenia by a doctor	Yes=1.00; no=0.00
**10**	Self-reported diagnosis of cancer by a doctor	Yes=1.00; no=0.00
**11**	Self-reported diagnosis of chronic kidney disease by a doctor	Yes=1.00; no=0.00
**12**	If you were walking on level ground with other healthy people of the same age, would you usually become short of breath or slow down because of chest discomfort?	Yes=1.00; no=0.00
**13**	How often do you have bowel movements each week?	< 3 times per week=1.00; other=0.00
**14**	During the past 12 months, did you have pain or discomfort in your body lasting ≥ 3 months that interfered with your life?	Yes=1.00; no=0.00
**15**	During the past 12 months, have you developed a frequent cough?	Yes, for ≥3 months=1.00; yes, for <3 months=0.50; no=0.00
**16**	Do you brush your teeth rarely or never, or have false teeth?	Yes=1.00; no=0.00
**17**	Physical activity in the past 12 months, including the usual type and duration of activities in occupational, commuting, domestic, and leisure time-related domains	Lowest quintile stratified by sex=1.00; other=0.00
**18**	During the past 12 months, have you lost weight (⩾2.5 kg) despite not trying to intentionally lose weight?	Yes=1.00; no=0.00
**19**	During the past 12 months, did you feel much sadder, or more depressed, than usual	Yes=1.00; no=0.00

	for ⩾2 weeks?	
**20**	How is your current general health status?	Poor=1.00**;** fair=0.50**;** good=0.50**;** excellent=0.00
**21**	Body mass index (kg/m^2)	< 18.5 or ≥ 28.0=1.00; ≥ 24.0 and < 28.0=0.50; ≥ 18.5 and < 24.0=0.00
**22**	Waist circumference (cm) to hip circumference ratio	≥ 0.95 for men or ≥ 0.90 for women=1.00; ≥ 0.90 and < 0.95 for men or ≥ 0.85 and < 0.90 for women=0.50; < 0.90 for men or < 0.85 for women=0.00
**23**	Measured heart rate, beats per min	< 60 or >100=1.00; ≥ 60 and ≤ 100=0.00
**24**	The ratio of forced expiratory volume in 1 s to the forced vital capacity measured to be < 0.7	Yes=1.00; no=0.00

eTable 2. Results of sensitivity analysis (i.e. excluding those diabetes cases within first 2 years)

Variables	Cases, n	Person-years	Incidence rate, per 10000 person-years	HR (95% CI)
Sleep quality index				
0	6,014	2281,678	26.4	1.00 (0.97 – 1.03)
1	6,605	1944,876	34.0	**1.08 (1.06 – 1.11)**
2	3,196	784,160	40.8	**1.16 (1.12 – 1.21)**
3	1,081	266,339	40.6	**1.11 (1.05 – 1.18)**
≥ 4	525	110,786	47.4	**1.28 (1.18 – 1.40)**
Sleep duration, hour				
≤ 5	1,587	415,166	38.2	1.03 (0.96 – 1.10)
6	2,591	760,581	34.1	1.00 (0.95 – 1.05)
7	4,385	1351,274	32.5	1.00 (0.96 – 1.04)
8	6,050	1981,170	30.5	1.02 (0.98 – 1.06)
9	1,960	605,840	32.4	1.04 (0.99 – 1.10)
≥ 10	848	273,808	31.0	1.10 (1.02 – 1.19)
Snoring				
None	7,197	2962,190	24.3	1.00 (0.97 – 1.03)
Sometimes	4,369	1299,973	33.6	1.03 (0.99 – 1.07)
Usually	5,855	1125,677	52.0	**1.18 (1.14 – 1.23)**
Daytime napping				
None	73,92	2136,364	34.6	1.00 (0.97 – 1.03)
Summer-only	6,510	2208,913	29.5	1.01 (0.97 – 1.05)
Usually	3,519	1042,562	33.8	**1.09 (1.05 – 1.15)**
Insomnia				
No	14,352	4518,735	31.8	1.00
Yes	3,069	869,105	35.3	1.01 (0.96 – 1.06)
Insomnia parameters				
Difficulty initiating sleep				
No	15,370	4789,373	32.1	1.00
Yes	2,051	598,467	34.3	1.03 (0.97 – 1.08)
Waking up too early				
No	15,398	4841,120	31.8	1.00
Yes	2,023	546,720	37.0	1.04 (0.99 – 1.10)
Daytime dysfunction				
No	17,012	5269,058	32.3	1.00
Yes	409	118,782	34.4	1.00 (0.90 – 1.11)
Sleep needing medicine				
No	17,190	5329,937	32.3	1.00

Yes	231	57,903	39.9	1.07 (0.94 – 1.22)

The data was analyzed using the model 4.
